# Developing a standardized list of entomological collection methods for use in databases

**DOI:** 10.3897/zookeys.861.32347

**Published:** 2019-07-08

**Authors:** Michael L. Ferro, Morgan Summerlin

**Affiliations:** 1 Clemson University Arthropod Collection, Department of Plant and Environmental Sciences, Clemson University, Clemson, SC 29634-0310, USA Clemson University Clemson United States of America; 2 Department of Art as Applied to Medicine, Johns Hopkins University School of Medicine, 1830 East Monument Street, Suite 7000, Baltimore, MD 21287-0022, USA Johns Hopkins University School of Medicine Baltimore United States of America

**Keywords:** curation, entomology, insect, museum

## Abstract

The current natural history specimen databasing paradigm focuses on standardizing occurrence data: where and when a specimen was collected. In order to gather more information about a particular species, researchers also must know how to encounter, and possibly collect, the species. For entomological specimens, collection method terminology written on labels has not been standardized, and perhaps should not be; however, use of a broad-scale collection method framework may aid in communication among researchers especially within the context of public databases. Three main categories of collection methods are proposed: active human collecting; active specimen orientation; and passive specimen collection and/or concentration. General categories contain more specific sub-categories and so on. A bibliography of useful works describing entomological collection and curation methods (e.g., “How to make an insect collection”) is provided.

## Introduction

For many invertebrates, distressingly little is known about their distribution, phenology, and natural history. Learning more about any aspect of a particular species requires three pieces of information: where to find it, when to go looking, and how to encounter it. For a select few species a meaningful encounter can take place without collecting the specimen, many dragonflies, bees, and butterflies can be sight identified, but for most species an accurate identification can be made only once a specimen is captured, after which it may be retained or released.

Recent technological advances and environmental regressions have precipitated a desire to create databases of natural history collections. To that end, Biodiversity Information Standards (TDWG) (www.tdwg.org), was created to “establish international collaboration among biological database projects” (https://www.tdwg.org/about/). One result was creation of the Darwin Core standard (http://rs.tdwg.org/dwc/), a p of agreed upon terms used when reporting the occurrence of taxa, the when and where.

However, little standardization has been applied to collection method (see “dwc:sampling Protocol” http://terms.tdwg.org/wiki/dwc:samplingProtocol. Examples include: “UV light trap”, “mist net”, and “Penguins from space: faecal stains reveal the location of emperor penguin colonies”). As more material is added to databases and more databases are available online, standardization of the collection method field will become important, otherwise useful collection methods may become lost within a fog of overly vague or overly specific individualized terminology resulting in reduced searchability and loss of ability to collate records based on collection method. Additionally, a better understanding of collection method possibilities may help expand methods used when attempting to control or eradicate pests, study endangered species, conduct comprehensive surveys, or develop efficient surveys. While Darwin Core’s terminology concerning collection method may or may not change, the entomological, and wider natural history community, should consider adopting a more standardized p of collection method terms and/or concepts to use when databasing physical specimens.

The universe is complicated and diverse. Often, to ease communication or reduce overwhelming complexity, humans will create a small set of “boxes” or categories that, together, hold the majority of the entities within a particular system. For example, the life stages of an insect can be separated into four general “boxes”: egg, larva, pupa, and adult. Despite a wide variety of exceptions (ametabolous and hemimetabolous orders, subimagos, paedomorphic females, hypermetamorphosis, prepupae, etc.) these are useful and meaningful designations. It is important to remember that these categories are for human convenience, may or may not follow the natural world closely, and may be more (or less) useful in different forms to different users. Therefore, attempts to refine concepts to create one system that works equally well in all situations may be foolhardy (like the search for a single species-concept, or the definition of “life”).

A general outline of collection methods is presented below with examples (Whitman et al. 2019 independently started a very similar p). Referencing each method would become cumbersome, therefore a bibliography of some popular, useful, and/or interesting sources on collecting terrestrial and aquatic arthropods is provided. Several qualifications should be interjected here: 1) the p may not be complete and can/will be added to over time, the design allows for easy expansion; 2) equally valid alternative arrangements of the general concepts may exist; 3) the p as presented is biased for finer divisions among popular methods (lights: UV, MV, LED), and more generalized for less widely-used methods (thermal lure); 4) “sampling,” a type of collecting, is used to capture a subset of a population often for statistical analysis and not dealt with in this scheme; 5) laboratory colonies are not ped below, presumably the progenitors were originally collected in one of the following manners.

## Collection methods

**Figure 1.**
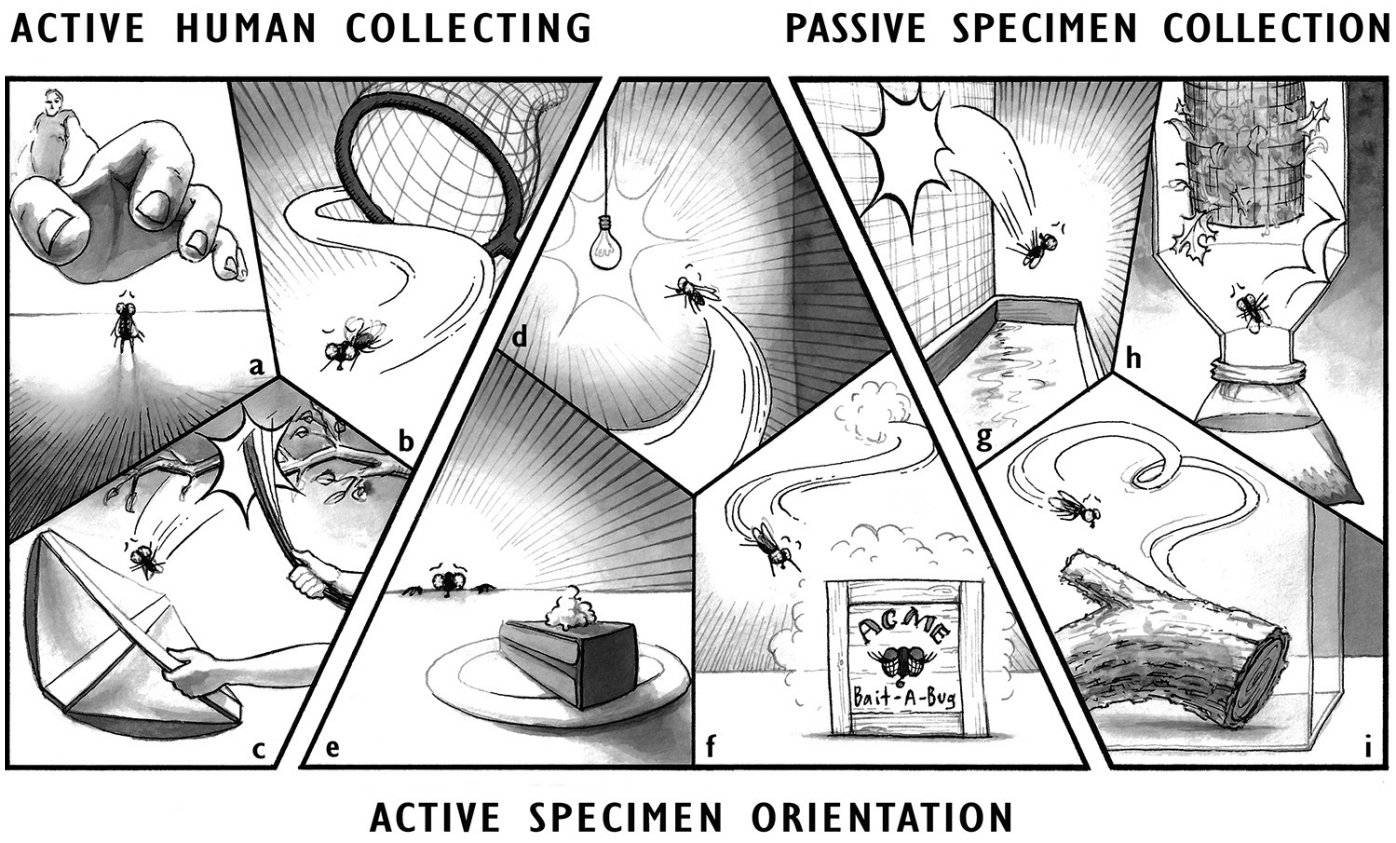
Examples of the three general collecting technique categories. Active human collecting: **a** hand collecting **b** netting **c** using a beat sheet; active specimen orientation **d** light trapping **e** baiting **f** pheromone attractant; passive specimen collection **g** flight intercept trap **h** Winkler Sampler **i** emergence trap.

All insect collecting falls into three broad categories: 1) active human collecting; 2) active specimen orientation; and 3) passive collection and/or concentration (Fig. 1).

### A. Active human collecting

Active human collecting falls within three different categories: immediate collection of a specimen from the environment; filtration of the environment to concentrate specimens; and agitation of specimens from their present location. In each case the collector is present, specimens are removed from a location where they (more or less) would naturally occur, and the collector has some knowledge of when and where a specimen was collected.

**1.** Immediate collection of specimens with hands, aspirator, vial, etc. While a specialized tool may be used to collect the specimens (e.g., aspirator), here, specimens are recognized as such, individually targeted, and often directly removed from their native substrate by the collector (contrast with “sifting” below which lacks many of these elements). The method provides the highest accuracy and precision when reporting the location or activities of the specimen (host plant/animal, substrate, etc.).

**1.1** Hand/Direct.

**2.** Sequestration and concentration of specimens using a net/strainer/filter. A net, sensu lato, is a filtering device. The meaningful difference among nets is not design (aquatic, aerial) but the size of the holes, as that determines the smallest organism likely to be captured. Entomologists rarely consider mesh size except in aquatic situations. However, the interplay of substrate sampled (which carries ecological information) and net design creates the categories most often used to define net type.

**2.1** Net (general).

**2.1.1** Net – Aquatic (from water).

**2.1.2** Net – Aerial (collection of flying specimens).

**2.1.3** Net – Sweeping (collection of specimens that were attached to a substrate).

**2.2** Vacuum collector (hand held or backpack device that gathers and filters substrate, may be air or liquid based).

**3.** Separation of specimens from a substrate or matrix, collector induced (not sequestered or concentrated, see below).

**3.1** Agitation (direct or indirect stimulus used to induce movement that increases likelihood of capture: flooding, soil flotation, waving hand over the trunk of a tree, sifting litter into a pan and immediately collecting, etc.).

**3.2** Beating (mechanical removal from substrate).

**3.3** Fogging (chemical used to cause agitation on, or removal from, substrate).

### B. Active specimen orientation

Specimens may be collected by altering the environment in such a way that the specimen actively moves toward the collection device or arena. Five general methods are employed: lights; chemicals; vibrations; heat; and ecological cues (often complex and utilize a combination of the preceding categories). Sections one through four below represent “attractants,” the factor that causes the specimen to reorient. They are often paired with a device from Category C when in use, for example a carrion baited (B.2.1) pitfall (C.1.1) trap. Often traps in section five are designed to combine an attractive feature and a passive collection method, for example a Lindgren funnel (B.5.2) incorporates an element of a flight intercept trap (C.1.2).

**1.** Positive phototaxis – specimen actively moves toward a light contrasted with darkness – night, caves, deep water. In general, this method is poorly known and little studied. The wavelengths of light differ among bulb type and are meaningful when attempting to recollect specimens and or understand specimen response to stimuli. Additional sub-categories could include specific wavelengths, wattages, etc. of each bulb type.

**1.1** Light (general, including standard visible-spectrum bulbs).

**1.1.1** Light – UV (general ultra-violet, typically fluorescent bulb).

**1.1.2** Light – MV (mercury vapor).

**1.1.3** Light – Metal halides.

**1.1.4** Light – LED (light emitting diode, presumably specific wavelengths/ranges could be reported).

**2.** Chemical bait/lure – use of a medium that emits chemicals to attract specimens. A natural division of this category would be autochthonous (self-originating) and allochthonous (other-originating). Mimicry of chemicals with synthetics, etc. would fall within the category of the chemical being mimicked.

**2.1** Bait – animal matter (carrion, dung, CO_2_) (allochthonous).

**2.2** Bait – plant matter (sugar, ethanol) (allochthonous).

**2.3** Bait – Pheromones, etc. (autochthonous) synthetic or natural, e.g. collection of Cupedidae with bleach or male Strepsiptera by using a live female as bait.

**3.** Vibrational lure – use of vibrations to attract specimens.

**3.1** Sound – air or substrate born vibrations (used for mosquitoes, Asian citrus psyllid).

**4.** Thermal lure – use of differences in temperature to attract specimens.

**4.1** Heat – used to attract mosquitoes and ticks.

**5.** Ecological cues – structural alteration of the environment that passively mimics or creates potential habitat or resources that could be used by the specimen. Often multiple attributes, such as color, shape, and size are required to create an effective lure/trap. Two equally valid ways of subdividing ecological cue collection methods are: 1) design (color, structure, etc.); or 2) resource being mimicked (food source, habitat, etc.). Generally, collection with ecological cues will be specific to taxa and an enormous variety of sub-sub-sub (e.g., 5.1.1) categories potentially exist. Plants excel in utilization of this category for pollination and seed dispersal. Often inclusion of chemicals, sounds, heat, etc. may render the trap more effective.

**5.1** Food resource trap – specimens orient toward the trap because it appears to represent a possible food source (yellow pan traps, trap crop, etc.).

**5.2** Habitat resource trap – specimens orient toward the trap because it appears to represent appropriate habitat (Lindgren funnel, bee “hotel”, purple panel trap, etc.).

**Alternative 5.1**:

Color trap – specimens actively orient towards the trap because of its color (yellow pan trap, purple panel trap, etc.).

**Alternative 5.2**:

Structure trap – creation of a seemingly suitable habitat (carpenter bee trap, Lindgren funnel, bee “hotel”, trap crop, etc.).

### C. Passive specimen collection and/or concentration

A structure is introduced into the environment, or an arena is created, where specimens, by virtue of their general movement, concentrate themselves in space and/or time.

**1.** Passive Trap – alteration of the environment in such a way that specimens unwittingly place themselves in a situation from which they cannot escape.

**1.1** Pitfall, ramp trap – gravity is used to collect and keep specimens in a location, essentially a “walking intercept trap”. The trap may be augmented with a barrier.

**1.2** Flight Intercept Trap (FIT), window trap – the path of flying insects is obstructed by a barrier. Specimen reaction to the barrier is often taxon specific, therefore placement of the collection device is meaningful. Height of the trap (ground based, canopy, etc.) may also be important.

**1.2.1** Malaise trap – specimens collected at top.

**1.2.2** Ground based FIT – specimens collected at bottom.

**1.2.3** Canopy Trap – elevated, often collection at top and bottom (Sante Trap).

**1.3** Flow intercept trap – insects in flowing water are obstructed by a barrier and waylaid or cannot escape.

**1.4** Suction trap – air flow is used to collect and keep specimens in a location.

**1.5** Glue – “sticky” material used to hold specimens in place (Tanglefoot® placed on a log or standing dead tree).

**2.** “Sifting” – Short term concentration and manipulation of inhabited material in such a way as to induce specimens to migrate to a collection point. The method is generally defined by the way the material is manipulated.

**2.1** Berlese/Tullgren funnel – habitat manipulation through heat (Berlese) or heat and light (Tullgren).

**2.2** Winkler sampler – habitat manipulation through increased surface area and drying.

**3.** Emergence – long term sequestration of inhabited material with the aim of allowing immature specimens to mature before collection. (As opposed to rearing. Rearing a specimen requires that you have already collected it, then provide it with resources and time to continue its life cycle.) Often the arena is designed so that emergent specimens concentrate themselves in a pitfall- or Malaise-type apparatus.

**3.1** Emergence chamber – material removed from original site, often fully enclosed.

**3.2** Emergence trap – full or partial enclosure of material in the field.

## Discussion

The above scheme allows for standardization of collection methods within and among databases. The ability to designate a collection method using general standard sets rather than specific, often regional, terms will aid in present and future communication. For example: “treading” and “flooding” are both A.3.1; “Brown sampler” and “vacuum benthos sampler” are both A.2.2; and “freshet,” “rejectamenta,” and “flood rubbish” are different terms for C.1.3 that occurs during a flood. For some methods separation of attractant used and method of capture will be important.

Additionally, researchers can build a better picture of which general collection methods are best at collecting specific taxa, the highest biodiversity, or even members within a particular guild. However, verbal descriptions can and should be maintained. For example, descriptions such as, “sifting pine duff,” “oak leaf litter” “Berlese Hemlock,” and “Berl. oak stump” all fall within the same collecting method, C.2, but all contain additional ecological information as well.

The creation and implementation of any standardized collection methods will require an extended discussion by the entomological and biodiversity databasing communities. The above p is provided to begin that discussion. Once approved, those standard methods can be provided as a drop-down p within databases. The numbering scheme can be retained to maintain organization and to illustrate relatedness of techniques, and verbal descriptions should be available to offer guidance.

The following is one possible implementation schema. Collection methods recorded on labels and within notes-sections of databases will continue to be as individualized, general, or specific as collectors wish, similar to the verbal locality on labels (e.g. 24 km SE Cole Camp). Within the database itself, a specific field would accept numbered methods. A drop-down p and descriptions can be provided. Multiple collection methods can be selected, similar to key-words for a manuscript. For example, specimens collected using an emergence chamber would get “C.3.1”; a CDC trap that emits CO_2_ and uses a UV light would be “B.1.1.1, B.2.1”. If the databasing community is so inclined, additional fields related to collection method can be added, such as killing agent, preservative, minimum and maximum time between specimen death and retrieval, etc.

While compiling the p above it became apparent that no single source contained descriptions of all collection methods known, even those which attempted to be as comprehensive as possible (e.g., Schauff 2001). Therefore, a bibliography is provided containing popular, useful, and/or interesting English language sources on collecting and preserving terrestrial and aquatic arthropods (see Bibliography and References, Suppl. material 1). Many of these works have gone through multiple editions, even though a single edition is cited. Some methods of collection and preservation have fallen out of favor or have been lost to the ages but are just as useful today as in the past, thus perusal of older literature is highly recommended.

However, two general areas need to be updated: chemical usage; and pinned insect labeling. The combination of the two sections below to the materials in the bibliography will go a long way toward elevating those references to a modern standard.

**Chemicals**: Many chemicals recommended in the past to kill or preserve specimens are now known to be deadly dangerous to humans, and more importantly, can damage the specimen DNA. Authors of new material, especially targeted at younger collectors, have removed dangerous chemicals from their works (modern 4-H manuals (Hall 1998) no longer recommend cyanide (Jones 1940)) and modern workers are more consciousness of killing and preservation methods. When utilizing, citing, or recommending older works, care should be taken to point out some chemical recommendations should not be followed. Consult Whitman et al. (2019) for a discussion on modern views of chemicals used for killing and preserving insects.

**Labeling**: Recent sources continue to repeat labeling recommendations that were created in a pre-personal printing era (e.g., Gibb and Oseto 2012). Those “systems” spread information across multiple labels (1: locality and date, 2: collector, 3: disposition of specimen) while today, all that information can be contained on a single label. Wheeler et al. (2001) provide a good description of label content and format (but not spacing). Modern specimen labeling should emphasize: 1) making sure that labels are positioned correctly laterally to reduce the specimen footprint and vertically to leave space for additional labels below; 2) keeping labels small and rectangular; and 3) making sure the label does not get bent or the hole enlarged. Correct labeling takes no more time than incorrect labeling and saves curators and workers an immense amount of work and frustration.

## Supplementary materials

Supplementary material 1 Bibliography Data type: reference p This dataset is made available under the Open Database License (http://opendatacommons.org/licenses/odbl/1.0/). The Open Database License (ODbL) is a license agreement intended to allow users to freely share, modify, and use this Dataset while maintaining this same freedom for others, provided that the original source and author(s) are credited.Michael L. Ferro, Morgan Summerlin
